# Diversity in Cortical Thymic Epithelial Cells Occurs through Loss of a Foxn1-Dependent Gene Signature Driven by Stage-Specific Thymocyte Cross-Talk

**DOI:** 10.4049/jimmunol.2200609

**Published:** 2023-01-01

**Authors:** Andrea J. White, Sonia M. Parnell, Adam Handel, Stefano Maio, Andrea Bacon, Emilie J. Cosway, Beth Lucas, Kieran D. James, Jennifer E. Cowan, William E. Jenkinson, Georg A. Hollander, Graham Anderson

**Affiliations:** * Institute of Immunology and Immunotherapy, University of Birmingham, Birmingham, United Kingdom;; † Department of Paediatrics and Institute of Developmental and Regenerative Medicine, University of Oxford, Oxford, United Kingdom;; ‡ Nuffield Department of Clinical Neurosciences, University of Oxford, Oxford, United Kingdom;; § Division of Infection and Immunity, University College London, London, United Kingdom;; ¶ Paediatric Immunology, Department of Biomedicine, University of Basel and University Children’s Hospital Basel, Basel, Switzerland; and; ‖ Department of Biosystems Science and Engineering, ETH Zurich, Basel, Switzerland

## Abstract

In the thymus, cortical thymic epithelial cells (cTECs) and medullary thymic epithelial cells support αβT cell development from lymphoid progenitors. For cTECs, expression of a specialized gene signature that includes *Cxcl12*, *Dll4*, and *Psmb11* enables the cortex to support T lineage commitment and the generation and selection of CD4^+^CD8^+^ thymocytes. Although the importance of cTECs in T cell development is well defined, mechanisms that shape the cTEC compartment and regulate its functional specialization are unclear. Using a *Cxcl12*^DsRed^ reporter mouse model, we show that changes in *Cxcl12* expression reveal a developmentally regulated program of cTEC heterogeneity. Although cTECs are uniformly *Cxcl12*^DsRed+^ during neonatal stages, progression through postnatal life triggers the appearance of *Cxcl12*^DsRed−^ cTECs that continue to reside in the cortex alongside their *Cxcl12*^DsRed+^ counterparts. This appearance of *Cxcl12*^DsRed−^ cTECs is controlled by maturation of CD4^−^CD8^−^, but not CD4^+^CD8^+^, thymocytes, demonstrating that stage-specific thymocyte cross-talk controls cTEC heterogeneity. Importantly, although fate-mapping experiments show both *Cxcl12*^DsRed+^ and *Cxcl12*^DsRed−^ cTECs share a common *Foxn1*^+^ cell origin, RNA sequencing analysis shows *Cxcl12*^DsRed−^ cTECs no longer express *Foxn1*, which results in loss of the FOXN1-dependent cTEC gene signature and may explain the reduced capacity of *Cxcl12*^DsRed−^ cTECs for thymocyte interactions. In summary, our study shows that shaping of the cTEC compartment during the life course occurs via stage-specific thymocyte cross-talk, which drives loss of *Foxn1* expression and its key target genes, which may then determine the functional competence of the thymic cortex.

## Key Points

- cTECs undergo progressive changes during postnatal life caused by loss of *Foxn1*.
- *Foxn1*^−^ cTECs remain within the cortex but lack cTEC signature genes.
- *Foxn1*^−^ cTECs show an impaired ability to influence developing thymocytes.

## Visual Abstract

**Figure:**
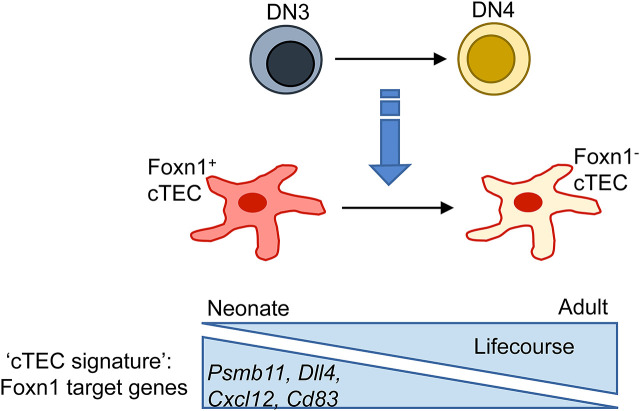

## Introduction

Self-tolerant MHC-restricted CD4^+^ and CD8^+^ αβT cells are produced exclusively in the thymus, a primary lymphoid organ that guides lymphoid progenitors through multiple developmental events. Importantly, many studies have shown the key roles that thymic stromal cells play in controlling thymocyte development (1–3). In particular, thymic epithelial cells (TECs) are functionally important during multiple developmental events that occur within anatomically distinct thymic areas (4). For example, EpCAM1^+^UEA1^+^Ly51^−^ medullary TECs (mTECs) are key in controlling T cell tolerance induction through the induction of both negative selection and Foxp3^+^ T cell development (5, 6). In contrast, cortex-resident cortical TECs (cTECs), typically defined as EpCAM1^+^ UEA1^−^ Ly51^+^ cells, are critical regulators of early T cell development. For example, on entry to the thymus, lymphoid progenitors undergo interactions with Delta-like 4 (DLL4)-expressing cTECs, which induce Notch signaling and direct progenitors toward a T cell fate (7–9). Immature thymocytes then transit through a series of CD4^−^CD8^−^ double-negative (DN) stages, including CD44^+^CD25^−^ DN1, CD44^+^CD25^+^ DN2, and CD44^−^CD25^+^ DN3, where they rearrange the *Tcrb* gene and express TCRβ protein as part of the cell-surface pre-TCR complex. Importantly, selection of TCRβ-expressing DN3 cells is also controlled by cTEC products, with CXCL12 and DLL4 acting in concert with the pre-TCR to generate large cohorts of preselection CD4^+^CD8^+^αβTCR^low^ thymocytes (10, 11). cTEC expression of MHC/self-peptide complexes then enables the cortex to support positive selection of CD4^+^CD8^+^ thymocytes that results in the generation of single-positive (SP) CD4^+^ and CD8^+^ thymocytes. In this study, the unique ability of cTECs to support positive selection is at least in part attributed to their specialized Ag-processing capabilities (12). For example, unique expression of *Psmb11*, the gene encoding the thymoproteosomal subunit β5t, enables cTECs to produce MHC class I (MHC I)–bound self-peptides that result in the effective positive selection of CD8^+^ thymocytes (13, 14). Similarly, cTEC expression of Cathepsin-L (15) and Prss16 (16) enables the generation MHC II/self-peptide complexes that drive efficient CD4^+^ thymocyte selection. Autophagic properties of cTECs may also aid in their control of positive selection (17). Significantly, many of the genes expressed by cTECs that underpin their functional specialization, including *Cxcl12*, *Dll4*, *Psmb11*, and *Ctsl*, are known targets of FOXN1 (18, 19), a transcription factor that plays an essential role in TEC development and function (20–22). Thus, cTEC expression of FOXN1 plays an important role in controlling a key gene expression signature that enables the cortex to support multiple stages of T cell development.

Despite this importance of cTECs for thymus function, our understanding of the mechanisms that control their development remains incomplete. To address cTEC development and heterogeneity, we examined Ly51^+^UEA1^−^ cTECs for evidence of heterogeneity using mice in which the fluorescent protein DsRed reports expression of the functionally important cTEC gene *Cxcl12* (23). We found that cTECs in adult mice can be readily subdivided into *Cxcl12*^DsRed+^ and *Cxcl12*^DsRed−^ subsets that both reside within the thymic cortex, with quantitative PCR (qPCR) analysis confirming their differential *Cxcl12* gene expression. Interestingly, examination of cTEC heterogeneity across the life course revealed a developmentally regulated program where cTECs were uniformly *Cxcl12*^DsRed+^ at neonatal stages, with *Cxcl12*^DsRed−^ cTECs appearing 1 wk after birth and persisting into adulthood. Importantly, whereas fate-mapping experiments show *Cxcl12*^DsRed+^ and *Cxcl12*^DsRed−^ cTECs both derive from FOXN1^+^ cells, RNA sequencing (RNA-seq) analysis showed these populations to be transcriptionally distinct. Unlike *Cxcl12*^DsRed+^ cTECs, *Cxcl12*^DsRed−^ cTECs lacked *Foxn1* expression, and this was accompanied by a change in the gene expression profiles of FOXN1 targets, including *Cxcl12* itself, as well as *Psmbl11*, and the Notch ligand *Dll4*. Furthermore, the emergence of *Cxcl12*^DsRed−^ cTECs was impaired in *Rag2*^−/−^, but not *Tcra*^−/−^, mice, and *Cxcl12*^DsRed−^ cTECs were impaired in their ability to form successful cellular interactions with thymocytes when compared with their *Cxcl12*^DsRed+^ counterparts. Taken together, our study identifies a developmentally regulated program of cTEC heterogeneity, where signals arising from the maturation of immature DN3 thymocytes cause transcriptional changes in the cTEC population that result in loss of *Foxn1* expression and transcripts of its downstream targets. This then creates epithelial heterogeneity in the thymic cortex that may influence functionality within the cTEC compartment.

## Materials and Methods

### Mice

The following mice on a C57BL/6 background were purchased from The Jackson Laboratory and used at 10 wk of age unless otherwise stated: *Cxcl12*^DsRed^ knockin (stock no. 022458) (23), which were used in isolation or crossed with *Tcr*α^−/−^ (stock no. 002116 (24), *Rag2*^−/−^(stock no. 008449) (25), *Foxn1*^Cre^ (stock no. 018448) (26), and Rosa26-stop-EYFP (stock no. 006148) (27). Control mice for experiments involving *Tcra*^−/−^ and *Rag2*^−/−^ mice were heterozygous littermate controls. RANK^Venus^ BAC transgenic mice were generated as described previously (28). Husbandry, housing, and experimental methods involving mice were performed at the Biomedical Services Unit at the University of Birmingham in accordance with the local Ethical Review Panel and U.K. Home Office Regulations (Animal project License no. P3ACFED06).

### Flow cytometry, cell sorting, and Abs

For TEC analysis, single-cell suspensions were generated by digesting thymic lobes with collagenase Dispase (2.5 mg/ml; Roche) and DNase 1 (40 mg/ml; Roche). CD45^−^ cells were enriched by the depletion of CD45^+^ cells using anti-CD45 beads and LS columns (Miltenyi Biotec). The following Abs were used for TEC analysis: anti-CD45 clone 30-F11 (eBioscience), anti-EpCAM1 clone G8.8 (eBioscience), anti-Ly51 clone 6C3 (BioLegend), anti–MHC II clone M5/114.15.2 (eBioscience), anti-CD80 clone 16-10A1 (BioLegend), CD104 clone 346-11A (BioLegend), and anti–MHC I 28-14-8. Biotinylated UEA-1 (Vector laboratories) was detected using streptavidin PECy7 (eBioscience). Cells were analyzed using a LSR Fortessa (Becton Dickinson) with data analysis carried out using FlowJo v10 (Becton Dickinson). For cell sorting, TEC subsets were identified using the earlier Abs and isolated using a FACSAria Fusion 1 cell sorter (Becton Dickinson). The sorting strategy for the different TEC subsets was as follows: Cxcl12^DsRed+^ cTEC, CD45^−^EpCAM1^+^UEA1^−^Ly51^+^CXCL12^DsRed+^; CXCL12^DsRed−^ cTEC, CD45^−^EpCAM1^+^UEA1^−^Ly51^+^CXCL12^DsRed−^; mTEC^lo^, CD45^−^EpCAM1^+^UEA1^+^Ly51^−^CD80^−^MHC II^−^; mTEC^hi^, CD45^−^EpCAM1^+^UEA^+^Ly51^−^CD80^+^MHC II^+^; CD104^+^ mTEC^lo^, CD45^−^EpCAM1^+^UEA1^+^Ly51^−^CD80^−^MHC II^−^CD104^+^; and CD104^−^ mTEC^lo^, CD45^−^EpCAM1^+^UEA1^+^Ly51^−^CD80^−^MHC II^−^CD104^−^.

### Immunohistochemistry and confocal microscopy

Thymus tissue from *Foxn1*^Cre^/*Rosa26*^YFP^/*Cxcl12* mice was isolated and fixed in 2% paraformaldehyde (PFA; Sigma) for 2 h, then overnight in 15% sucrose (Sigma). Thymic lobes were frozen on dry ice and sectioned at 7 μm within 24 h of freezing. eYFP protein in sections from *Foxn1*^Cre^/*Rosa26*^YFP^/*Cxcl12*^DsRed^ was amplified using rabbit anti-GFP (ThermoFisher) and donkey anti-rabbit 488 (ThermoFisher). Sections were counterstained with DAPI (Sigma) and mounted using Prolong Diamond (ThermoFisher). Sections were imaged using Zeiss Zen 880 microscope (Zeiss) and analysis using Zeiss Zen Black (Zeiss).

### qPCR analysis

Real-time PCR was performed as described previously (29) on a Corbett Rotor Gene-3000 PCR machine (Qiagen) using a SensiMix SYBR No ROX Kit (Meridian Bioscience-Bioline) and primers specific for *Actb* (β-actin) (Qiagen) and indicated genes of interest (Sigma-Merck). Data shown are typical of at least two independently sorted sample sets; histograms represent the mean (± SEM) of replicate reactions. Primer sequences used were: *Foxn1*, forward 5′-*CAAATTCTGCAGGGGTCAGA*-3′ and reverse 5′-*TGGGGTGCAATCCTCTGATA*-3′; *Cxcl12*, forward 5′-*GCTCTGCATCAGTGACGGTA*-3′ and reverse 5′-*TGTCTGTTGTTGTTCTTCAGC*-3′; *Psmb11*, forward 5′-*ATCGCTGCGGCTGATACTC*-3′ and reverse 5′-*GCAGGACATCATAGCTGCCAA*-3′; *Prss16*, forward 5′-*GTATTTCTGCACATAGGAGGCG*-3′ and reverse 5′-*TGTTCTAGGCTTATCACCAGGG*-3′; *Cd83*, forward 5′-*AGGGCCTATTCCCTGACGAT*-3′ and reverse 5′-*CTTCCTTGGGGCATCCTGTC*-3′; *Dll4*, forward 5′-*GAAGCGCGATGACCACTTCG*-3′ and reverse 5′-*TGGACGGCAGATGCACTCAT*-3′; *Ly75*, forward 5′-*GCTCAGGTAATGATCCATTCACC*-3′ and reverse 5′-*TTAGTTCCGCTACAGTCCTGG*-3′; *Ctsl*, forward 5′-*ATCAAACCTTTAGTGCAGAGTGG*-3′ and reverse 5′-*CTGTATTCCCCGTTGTGTAGC*-3′; *Epcam1*, forward 5′-*TTGCTCCAAACTGGCGTCTAA*-3′ and reverse 5′-*GCAGTCGGGGTCGTACA*-3′; *Aire*, forward 5′-*TGCATAGCATCCTGGACGGCTTCC*-3′ and reverse 5′-*CCTGGGCTGGAGACGCTCTTTGAG*-3′; *Trpm5*, forward 5′-*CCAGCATAAGCGACAACATCT*-3′ and reverse 5′-*GAGCATACAGTAGTTGGCCTG*-3′; *Ccl21a*, forward 5′-*ATCCCGGCAATCCTGTTCTC*-3′ and reverse 5′-*GGGGCTTTGTTTCCCTGGG*-3′; and *Actb* (β-actin), QuantiTect Mm *Actb* 1SG Primer Assay (QT00095242; Qiagen).

### Bulk RNA-seq

RNA samples were extracted using the Qiagen RNeasy kit. Libraries were prepared using the SMARTer Ultra Low Input RNA Kit for Sequencing as per the manufacturer’s instructions and sequenced on an Illumina NovaSeq platform. Reads were trimmed for adapter contamination using Trimmomatic (version 0.36) and aligned to the mm10 mouse genome using STAR (version 2.7.3a) (30, 31). Reads were assigned to genes using HTSeq (version 0.12.4) with the option “intersection-nonempty” (32). Differentially expressed genes were identified using edgeR (false discovery rate < 0.05) (33). Enrichment of *Foxn1* high-confidence genes (18) was assessed by comparing the log_2_-fold expression for *Foxn1* high-confidence genes with a control set of genes matched by expression decile using a Wilcoxon rank sum test. Sequencing data are available at the Gene Expression Omnibus (GEO; accession number GSE205940, https://www.ncbi.nlm.nih.gov/geo/query/acc.cgi?acc=GSE205940). Gene ontology analysis was performed using clusterProfiler (34).

### Cell conjugate analysis

Thymocyte–TEC conjugate experiments were carried out using a protocol adapted from Hare et al. (35). In short, CD45^−^EpCAM1^+^ TECs were FACS sorted from 10-wk-old *Cxcl12*^DsRed^ mice and neonatal day 2 wild-type (WT) mice and labeled with CFSE according to the manufacturer’s instructions (ThermoFisher). A single-cell suspension of WT adult thymocytes was labeled with CellTrace Violet according to the manufacturer’s instructions (ThermoFisher), and the two cell types were mixed at a 5:1 ratio (thymocytes:TEC). The mixed suspension was then centrifuged, the supernatant removed, and the cell pellet vortexed and incubated at 37°C for 20 min, a time point that enables successful conjugate formation between WT TECs and thymocytes (35). Samples were resuspended in a volume of 200 μl of PBS (Sigma) and analyzed using a BD LSR Fortessa.

## Results

### Progressive loss of *Cxcl12* expression identifies a developmentally regulated program of cTEC heterogeneity

**FIGURE 1.**
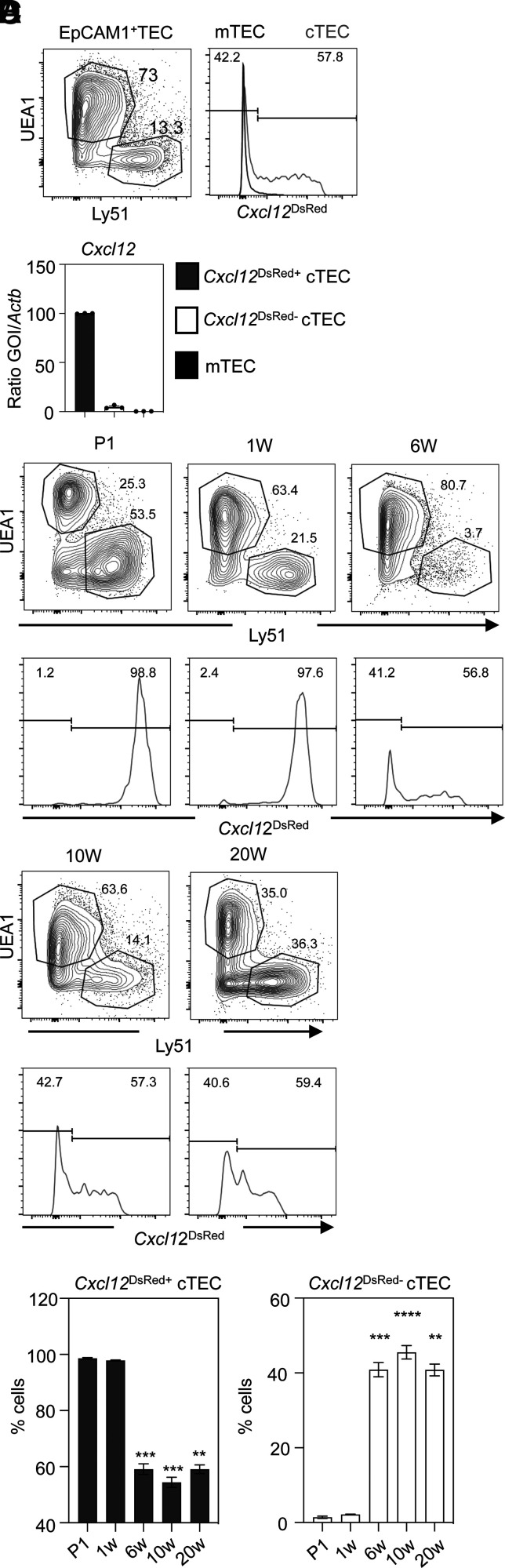
*Cxcl12* expression defines developmentally controlled heterogeneity in cTECs. (**A**) Flow cytometric analysis of EpCAM1^+^CD45^−^ TECs from adult 10-wk-old *Cxcl1*2^DsRed^ mice, separated into UEA1^+^Ly51^−^ mTECs and UEA1^−^Ly51^+^ cTECs. Levels of *Cxcl12*^DsRed^ expression in cTECs (gray line) and mTECs (black line) are shown. (**B**) qPCR expression of *Cxcl12* mRNA in FACS-sorted *Cxcl12*^DsRed+^ and *Cxcl12*^DsRed−^ cTEC subsets, with mTECs shown for comparison. (**C**) Time-course analysis of *Cxcl12*^DsRed^ expression in cTECs, identified using the gating shown, as UEA1^−^Ly51^+^ cells, from mice at indicated ages. Gates are set using mTECs as in (A). (**D**) Quantitation of *Cxcl12*^DsRed+^ and *Cxcl12*^DsRed−^ cTEC subsets. Each time point is from a minimum of *n* = 4 mice and at least three separate experiments: P1, *n* = 5; 1 wk (1W), *n* = 4; 6W, *n* = 5; 10W, *n* = 9; and 20W, *n* = 6. The *p* values are as follows and indicate the significance relative to P1, using a Mann–Whitney nonparametric test: **p < 0.01, ***p < 0.001, ****p < 0.0001. Error bars represent mean ± SEM.

In the thymus, cTECs are classically defined as the Ly51^+^UEA1^−^ subset of EpCAM1^+^ TECs. Although the functional properties of cTECs are well described, relatively little is known about the cellular and molecular interactions that control their development and potential functional heterogeneity. To investigate this, we made use of *Cxcl12*^DsRed^ reporter mice (23) in which DsRed expression identifies cells expressing *Cxcl12*, a cTEC-expressed chemokine that is an important regulator of thymocyte migration and development. Surprisingly, flow cytometric analysis of Ly51^+^UEA1^−^ cTECs from 10-wk-old adult *Cxcl12*^DsRed^ mice revealed striking heterogeneity with regard to DsRed expression, with the presence of distinct subsets of *Cxcl12*^DsRed+^ and *Cxcl12*^DsRed−^ cTECs (Fig. 1A). Importantly, when FACS-purified DsRed^+^ and DsRed^−^ cTEC cells were analyzed for *Cxcl12* mRNA expression by qPCR, we saw that the abundant expression of *Cxcl12* mRNA in DsRed^+^ cells was lacking in DsRed^−^ cells (Fig. 1B). Thus, heterogeneity in adult cTECs described in this article reflects true heterogeneity in their *Cxcl12* expression and is not merely a feature of DsRed reporter expression.

To examine cTEC heterogeneity further, we performed time-course analysis from birth up to 20 wk of adulthood. Interestingly, we saw that cTECs from neonatal (postnatal day 1 [P1]) mice were uniformly *Cxcl12*^DsRed+^ (Fig. 1C). Although the vast majority of cTECs were also Cxcl12^DsRed+^ at the 1-wk stage, we detected a distinct *Cxcl12*^DsRed−^ cTEC subset at 6 wk of life (Fig. 1C), with the proportions of *Cxcl12*^DsRed+^ and *Cxcl12*^DsRed−^ cTECs remaining constant for the remainder of the observation period (Fig. 1C, 1D). Collectively, these findings identify *Cxcl12*^+^ and *Cxcl12*^−^ subsets within the bulk cTEC compartment that are ordered in their appearance during development, suggesting the cTEC compartment undergoes developmentally regulated changes that can be measured by differences in *Cxcl12* expression.

### *Cxcl12*^DsRed−^ cTECs are transcriptionally distinct from their *Cxcl12*^DsRed±^ counterparts and lack *Foxn1* expression and a FOXN1-dependent gene signature

**FIGURE 2.**
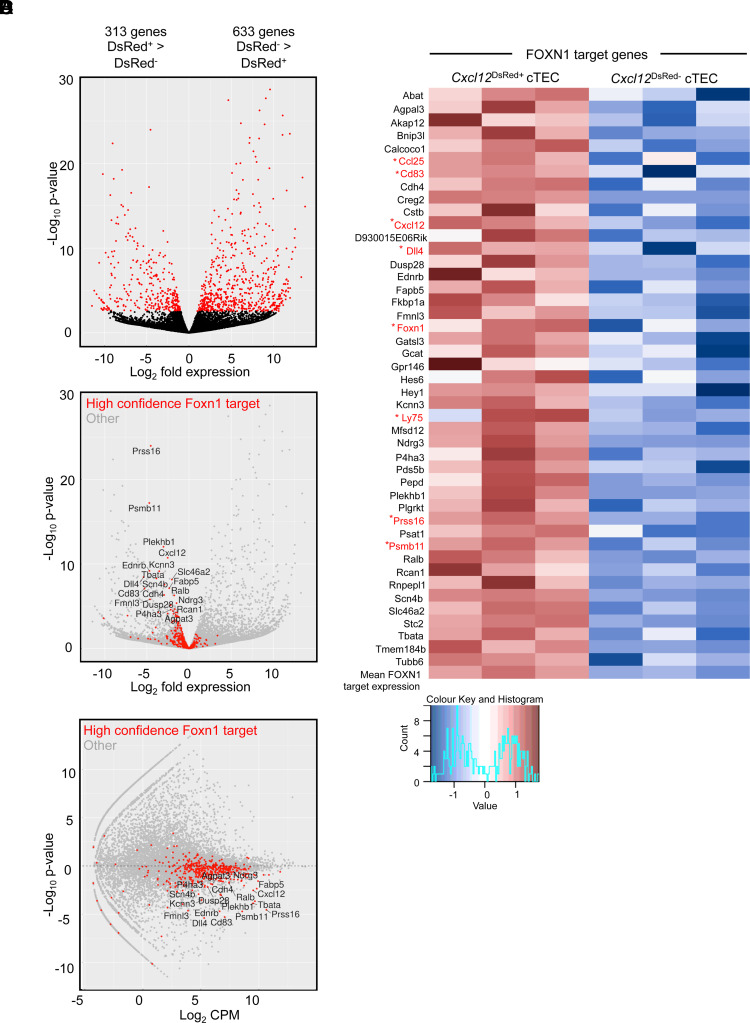
*Cxcl12*^DsRed+^ and *Cxcl12*^DsRed−^ cTEC subsets are transcriptionally distinct. RNA-seq analysis of FACS-sorted *Cxcl12*^DsRed+^ and *Cxc*l*12*^DsRed−^ cTECs from 10-wk-old *Cxcl12^DsRed^* mice. (**A**) A volcano plot of differentially expressed genes between cTEC subsets; red dots represent false discovery rate < 0.05, and black dots represent no significance. (**B**) A volcano plot of differentially expressed genes, emphasizing significant FOXN1 high-confidence target genes shown by red dots, with all other genes represented by gray dots. (**C**) A plot of log-intensity ratios (*M*-values) versus log-intensity averages (*A*-values) for all genes highlighting high-confidence FOXN1 target genes in red; the other genes are shown in gray. Graphs show a technical triplicate of a single experiment that is representative of three individually sorted biological replicates. (**D**) A heatmap of significantly differentially expressed FOXN1 target genes as identified in Žuklys et al. (18) and scaled mean expression of all FOXN1 target genes. Only FOXN1 targets with mean expression >1 count per million (CPM) were included. Genes associated with cTEC phenotype/function are highlighted in red.

**FIGURE 3.**
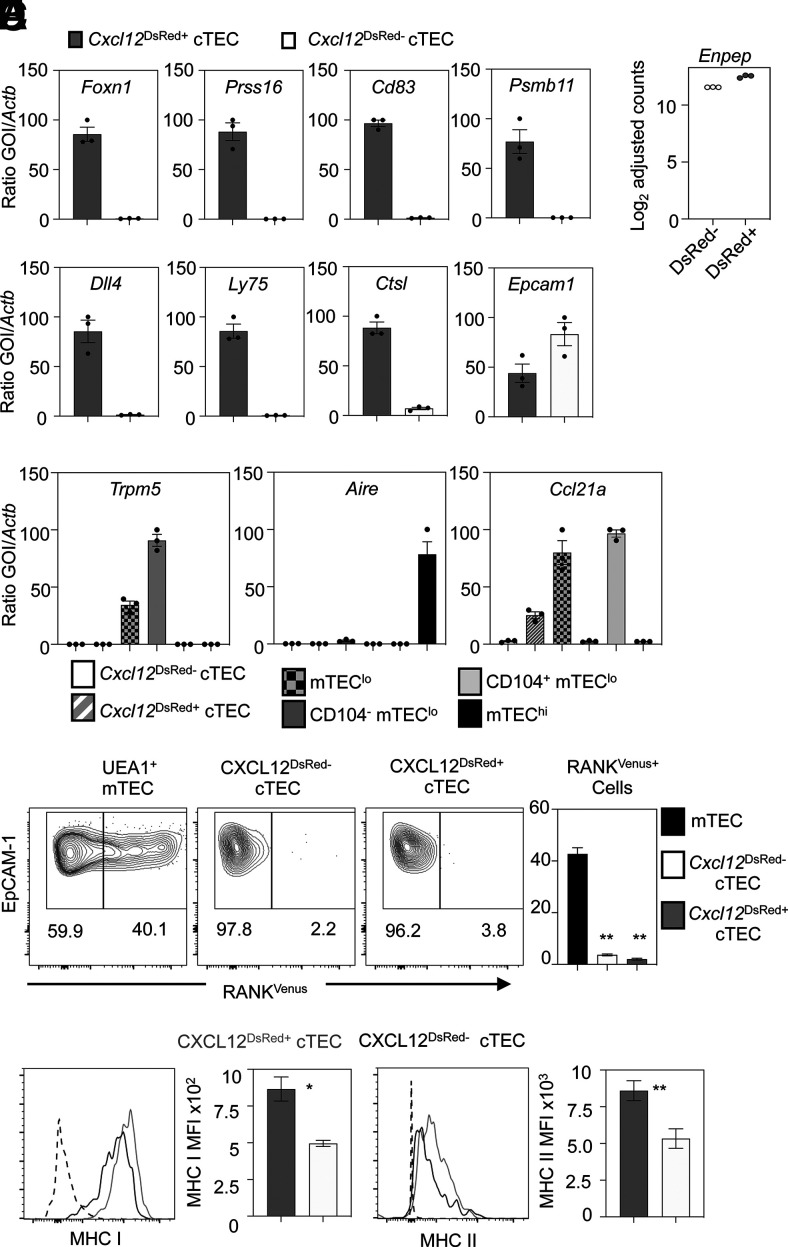
*Cxcl12^DsRed^*^−^ cTECs lack expression of *Foxn1* and a FOXN1 target gene signature. (**A**) Analysis of gene expression by qPCR in *Cxcl12*^DsRed+^ (gray bars) and *Cxcl12*^DsRed−^ (white bars) cTECs that were FACS sorted from 10-wk-old *Cxcl12^DsRed^* mice. (**B**) Levels of expression of *Enpep* obtained from bulk RNA-seq data in *Cxcl12*^DsRed+^ and *Cxcl12*^DsRed−^ cTECs. (**C**) qPCR analysis of mTEC-expressed genes *Trpm5*, *Aire*, and *Ccl21a* in *Cxcl12*^DsRed+^ and *Cxcl12*^DsRed−^ cTECs compared with relevant mTEC subsets. For all qPCRs, graphs represent data obtained from at least two independently sorted biological samples, with dots showing technical repeats. Error bars represent mean ± SEM. (**D**) Flow cytometric analysis of RANK^Venus^ expression by total UEA1^+^ mTECs, and *Cxcl12*^DsRed+^ and *Cxcl12*^DsRed−^ cTECs from Cxc12^DsRed^RANK^Venus^ reporter mice; *n* = 5 from five separate experiments. (**E**) Flow cytometric analysis of indicated cell-surface markers in *Cxcl12*^DsRed+^ (gray line) and *Cxcl12*^DsRed−^ (black line) cTECs from 10-wk-old Cxcl12^DsRed^ mice. Control staining levels obtained via omission of primary Abs are shown as a gray line. (E) MFI analysis of indicated markers in *Cxcl12*^DsRed^ cTEC subsets is also shown. Data are from at least three experiments; for MHC II, *n* = 8; MHC I, *n* = 4. The *p* values are as follows and indicate the significance relative to P1, using a Mann–Whitney nonparametric test: *p < 0.05, **p < 0.01. Error bars represent mean ± SEM.

To understand the events underlying this cTEC heterogeneity, we used RNA-seq to compare the transcriptomes of *Cxcl12*^DsRed+^ and *Cxcl12*^DsRed−^ cTECs. In this study, *Cxcl12*^DsRed+^ and *Cxcl12*^DsRed−^ subsets of total CD45^−^EpCAM1^+^UEA1^−^Ly51^+^ cTECs were FACS sorted from 10-wk-old adult *Cxcl12*^DsRed^ reporter mice, with experiments performed in triplicate to produce three independent biological replicates for each subset. This approach identified 946 genes differentially expressed between DsRed^+^ and DsRed^−^ cTECs (Fig. 2A). Much of this transcriptomic difference was driven by the lower expression of genes known to be direct targets of FOXN1 in *Cxcl12*^DsRed−^ cTECs relative to *Cxcl12*^DsRed+^ cTECs, and this correlated with the lack of expression of *Foxn1* in the former (*p* < 0.0001, Wilcoxon rank sum test; (Fig. 2B–D) (18). For example, the heatmap analysis in (Fig. 2D shows clear differences in expression of *Foxn1* and several of its direct targets, including *Cxcl12*, *Dll4*, *Cd83*, *Ccl25*, *Ly75*, *Psmb11*, and *Prss16.* Further qPCR analyses confirmed data obtained from RNA-seq experiments, including the absence of *Foxn1* transcripts in *Cxcl12*^DsRed−^ cTECs (Fig. 3A), as well as the absence of transcripts encoding FOXN1 target genes that play key roles in specific stages of thymocyte development, including thymocyte migration (*Cxcl12*), Notch signaling (*Dll4*), and Ag processing/presentation (*Prss16*, *Psmb11*, *Ctsl*, *Ly75*). By contrast, *Cxcl12*^DsRed+^ and *Cxcl12*^DsRed−^ cTEC subsets showed no reduction in levels of *Epcam1* mRNA (Fig. 3A). Importantly, *Cxcl12*^DsRed+^ and *Cxcl12*^DsRed−^ cTECs showed comparable levels of *Enpep* expression, the gene encoding the cTEC marker Ly51 (Fig. 3B). qPCR analysis showed both *Cxcl12*^DsRed+^ and *Cxcl12*^DsRed−^ cTEC subsets lacked expression of mTEC markers, including the tuft cell marker *Trpm5*, as well as *Aire* and *Ccl21a* that were readily detectable within mTEC subsets (Fig. 3C). Moreover, by crossing *Cxcl12*^DsRed^ with RANK^Venus^ reporter mice, we saw both cTEC subsets lacked expression of RANK, a key marker and regulator of mTECs (Fig. 3D). These findings support the idea that *Cxcl12*^DsRed−^ Ly51^+^UEA1^−^ cells belong to the cTEC lineage and do not contain mTEC lineage cells. Finally, although both *Cxcl12*^DsRed+^ and *Cxcl12*^DsRed−^ cTECs expressed MHC I and MHC II, their cell-surface expression levels were significantly lower on *Cxcl12*^DsRed−^ cTECs (Fig. 3E).

**FIGURE 4.**
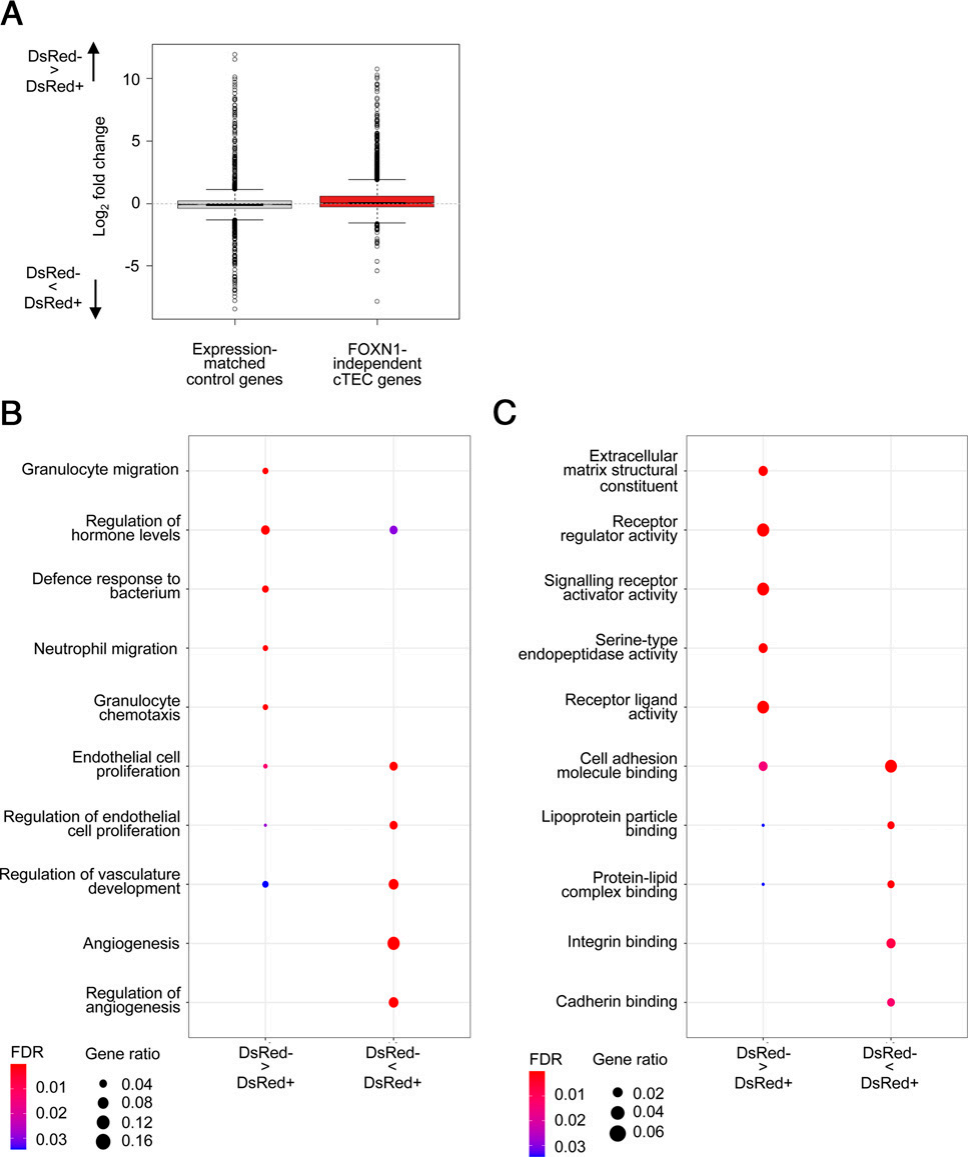
Comparative analysis of gene expression in *Cxcl12*^DsRed+^ and *Cxcl12^DsRed^*^−^ cTECs. (**A**) Boxplot of Foxn1-independent cTEC gene expression and genes matched by decile expression. cTEC marker genes were defined as those expressed more highly in perinatal or mature cTECs than other cell types in a reference dataset (36). cTEC markers that were Foxn1 enhanced [significantly upregulated or ≥0.25 log_2_-fold higher with increased Foxn1 (18)] were removed to leave only FOXN1-independent cTEC markers. Expression of FOXN1-independent cTEC markers was similar between *Cxcl12*^DsRed+^ and *Cxcl12*^DsRed−^ cTECs. (**B** and **C**) Dot plots of gene ontology analysis for biological processes (B) and molecular functions (C) are shown.

To examine further the nature of *Cxcl12*^DsRed−^ cTECs in relation to their *Cxcl12*^DsRed+^ counterparts, we searched for genes that were differentially expressed between the two subsets (Supplemental Table I). When we analyzed the expression of cTEC marker genes (36), removing those known to be FOXN1 dependent (18), we saw the expressions of cTEC marker genes in *Cxcl12*^DsRed+^ and *Cxcl12*^DsRed−^ cTEC subsets were similar (Fig. 4A). Interestingly, however, gene ontology analysis pointed toward some potential differences. For example, in *Cxcl12*^DsRed+^ cTECs, we saw enrichment of pathways associated with regulation of endothelial cell proliferation, angiogenesis, and vascular development, whereas *Cxcl12*^DsRed−^ cTECs showed enrichment of other distinct pathways, including serine-type endopeptidase activity regulation of granulocyte migration (Fig. 4B, 4C). Collectively, these data suggest that although the major difference between *Cxcl12*^DsRed+^ and *Cxcl12*^DsRed−^ cTECs relates to expression of *Foxn1* and a FOXN1-dependent cTEC signature, they may also harbor gene expression patterns that point toward functional differences between the two subsets.

**FIGURE 5.**
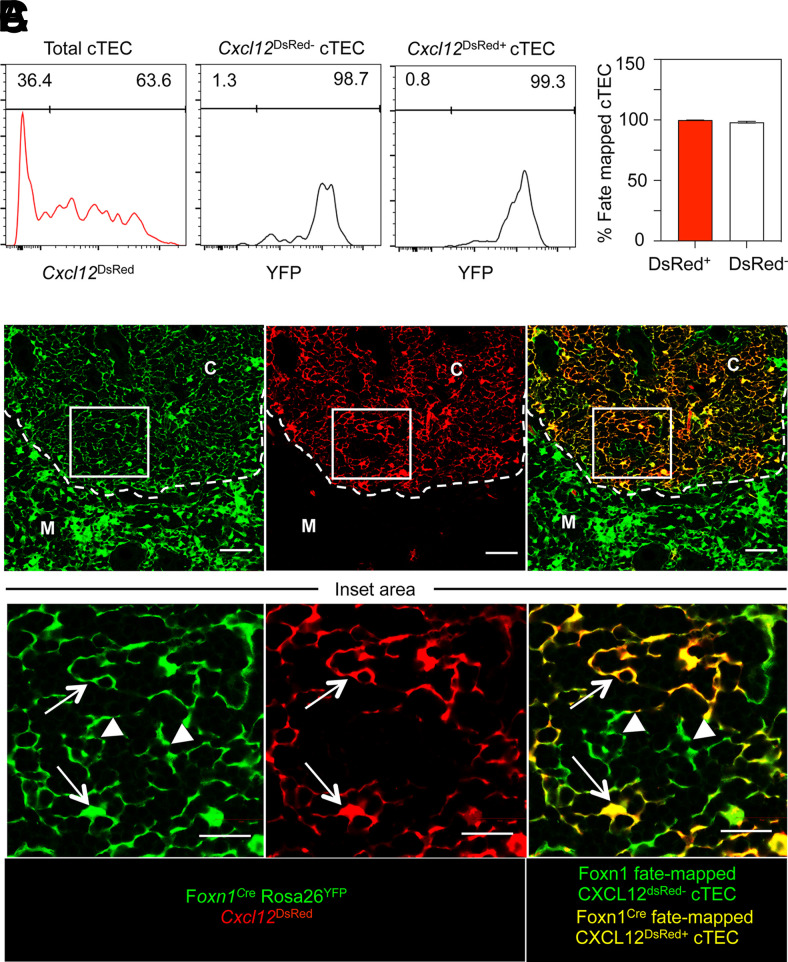
Both *Cxcl12*^DsRed±^ and *Cxcl12*^*DsRed*−^ cTECs are derived from Foxn1-expressing cells. (**A**) Gating for the identification of *Cxcl12^DsRed+^* and *Cxcl12*^*DsRed*−^ cTECs in 10-wk-old *Foxn1*^Cre^/Rosa26-YFP/*Cxcl12*^DsRed^ mice is shown, as well as levels of YFP expression in these cells, where YFP indicates a history of Foxn1 expression. Gates are set following gating on YFP levels in CD45^+^ cells, where *Foxn1*^Cre^-mediated fate mapping is absent. Quantitation is shown in (**B**). Data are from five mice across three experiments. (**C**) Confocal analysis of PFA-treated thymus sections from *Foxn1*^Cre^/Rosa26RYFP/*Cxcl12^DsRed^* mice, analyzed for expression of YFP (shown in green) and DsRed (red), with coexpression appearing yellow. Upper panels are ×10 original magnification and show cortex (C) and medulla (M) areas defined by DAPI; dotted line is the corticomedullary junction. Scale bars, 50 μm. The boxed area highlighted in the upper panels represents an area of the cortex that is shown at ×40 original magnification in the image row below. Scale bars in the lower images represents 20 μm. Arrows identify *Foxn1*Cre fate-mapped YFP^+^*Cxcl12^DsRed+^* cTECs, while arrowheads identify *Foxn1*Cre-fate mapped YFP^+^*Cxcl12^DsRed^*^−^ cTECs. Images are examples of four sections randomly chosen from four separate mice across two separate experiments.

The presence of *Foxn1*^−^ cTECs in the adult thymus could occur as a result of the downregulation of FOXN1 in cells that had previously expressed FOXN1, or via the progressive emergence of a cTEC subset with no prior history of FOXN1 expression. To distinguish between these possibilities, we used a fate-mapping approach to examine the history of FOXN1 expression in *Cxcl12*^DsRed+^ and *Cxcl12*^DsRed−^ cTECs. In adult *Foxn1*^Cre^/*Rosa26*^YFP^/*Cxcl12*^DsRed^ mice, the vast majority of both *Cxcl12*^DsRed+^ and *Cxcl12*^DsRed−^ cTECs were *Foxn1*^Cre^ fate mapped (Fig. 5A, 5B), indicating both cTEC subpopulations were generated from *FOXN1*-expressing cells. Confocal analysis of thymus sections from these mice demonstrated that both *Cxcl12*^DsRed+^ and *Cxcl12*^DsRed−^ *Foxn1^Cre^* fate-mapped cells were present within thymic cortex areas (Fig. 5C). Use of confocal microscopy to further examine the phenotypic properties of cortex-resident *Cxcl12*^DsRed−^ cells was unfortunately hampered by the impact of PFA fixation, required to preserve DsRed protein, on successful Ab staining. Collectively, these findings show *FOXN1* is not uniformly expressed within the adult cTEC compartment, with the presence of *FOXN1*^−^ cTECs providing an explanation for the presence of those cells that lack expression of the target gene *Cxcl12*. Importantly, our findings also show that heterogeneity in FOXN1 expression by cTEC extends beyond differences in *Cxcl12* expression and includes the differential expression of FOXN11-controlled loci (e.g., *Dll4*, *Ccl25*, *Psmbl1*, *Prss16*) that are important in the regulation of cortical T cell development. Despite this change in the cTEC-specific mRNA signature, *Cxcl12*^DsRed−^ cTECs continue to reside within cortical areas alongside their *Cxcl12*^DsRed+^ counterparts, where they contribute to the reticular epithelial network of the adult thymic cortex.

### Stage-specific thymocyte cross-talk regulates cTEC heterogeneity

**FIGURE 6.**
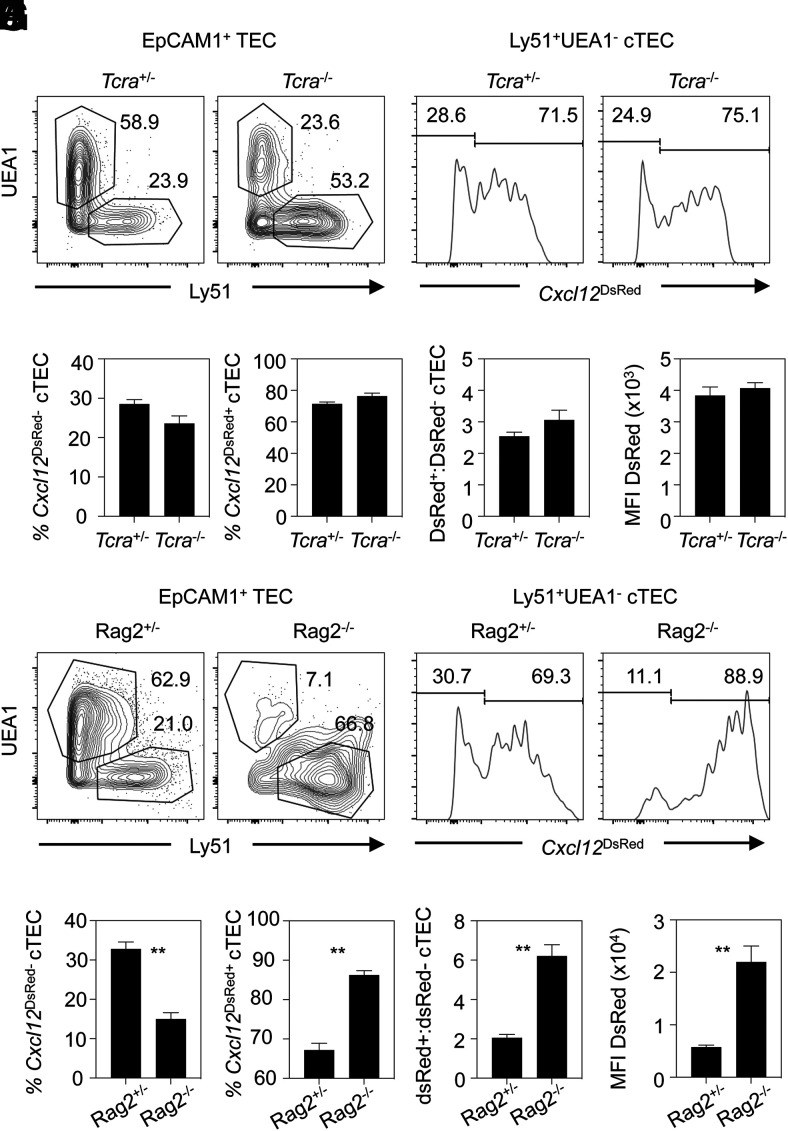
Stage-specific thymocyte cross-talk controls cTEC heterogeneity. (**A**) Identification of cTECs and mTECs in 10-wk-old *Cxcl12^DsRed^*/*Tcra*^−/−^ mice and *Cxcl12^DsRed^*/*Tcra*^+/−^ littermate controls, with (**B**) showing levels of *Cxcl12*^DsRed^ expression after gating on cTECs. (**C** and **D**) Percentages (C) and ratios (D) of *Cxcl12*^DsRed+^ and *Cxcl12*^DsRed−^ cTECs in *Tcra*^−/−^ and *Tcra*^+/−^ mice are shown alongside MFI of DsRed in cTEC subsets (**E**). (**F**–**J**) Similar analysis of *Cxcl12*^DsRed^/*Rag2*^−/−^ mice and *Cxcl12*^DsRed^/*Rag2*^+/−^ littermate controls. All data are representative of at least three independent experiments, using the following numbers of mice: *Cxcl12*^DsRed^/*Tcra*^−/−^, *n* = 12; *Cxcl12*^DsRed^/*Tcra*^+/−^, *n* = 10; *Cxcl12*^DsRed^/*Rag2*^−/−^, *n* = 6; *Cxcl12*^DsRed^/*Rag*^+/−^, *n* = 6. The *p* values indicate significance using a Mann–Whitney nonparametric test: **p < 0.01. Error bars represent mean ± SEM.

Signals from developing thymocytes are known to regulate the development and formation thymic microenvironments, a process termed thymic cross-talk (37, 38). Much of our understanding of this process comes from studies examining the cellular interactions that govern events in the thymus medulla. For example, cross-talk with mTEC regulates development of Aire^+^ mTECs (39, 40) and post-Aire stages (29, 41). In contrast, how thymic cross-talk signals influence the thymic cortex, and in particular how they might control the Cxcl12/Foxn1 cTEC heterogeneity described in this article, is unclear. To examine this specific aspect, we crossed *Cxcl12*^DsRed^ mice with *Rag2*^−/−^ and *Tcra*^−/−^ mice, where T cell development is blocked at the CD4^−^CD8^−^ or CD4^+^CD8^+^ stages, respectively. Interestingly, in *Tcra*^−/−^*Cxcl12*^DsRed^ mice, cTEC heterogeneity was comparable with littermate controls (Fig. 6A, 6B), with no alterations in the proportions of *Cxcl12*^DsRed+^ and *Cxcl12*^DsRed−^ cTECs (Fig. 6C) or the ratio of DsRed^+^:DsRed^−^ cTECs (Fig. 6D). Mean fluorescence intensity (MFI) levels of DsRed in *Cxcl12*^DsRed+^ cTECs were also comparable (Fig. 6E). Thus, the appearance of *Cxcl12*^DsRed−^ cTECs occurs normally in the absence of CD4^+^ and CD8^+^ SP thymocytes, suggesting that positive selection of CD4^+^CD8^+^ thymocytes is not essential for the generation of *Cxcl12*^DsRed^ cTEC heterogeneity. In contrast, when we performed similar analysis of *Rag2*^−/−^*Cxcl12*^DsRed^ mice (Fig. 6F), we saw that the proportion of *Cxcl12*^DsRed−^cTECs was decreased, with a concomitant increase in *Cxcl12*^DsRed+^ cTECs (Fig. 6G, 6H). This finding was accompanied by a skewing of the DsRed^+^:DsRed^−^ cTEC ratio in favor of DsRed^+^ cells (Fig. 6I), with *Cxcl12*^DsRed+^ cTECs in *Rag2*^−/−^ mice also showing higher levels of DsRed compared with littermate controls (Fig. 6J). These findings show that in the absence of CD4^+^CD8^+^ thymocytes, the appearance of *Cxcl12*^DsRed−^ cTECs is impaired, suggesting that maturation of CD4^−^CD8^−^ thymocytes is an important regulator of cTEC heterogeneity in the adult thymus.

**FIGURE 7.**
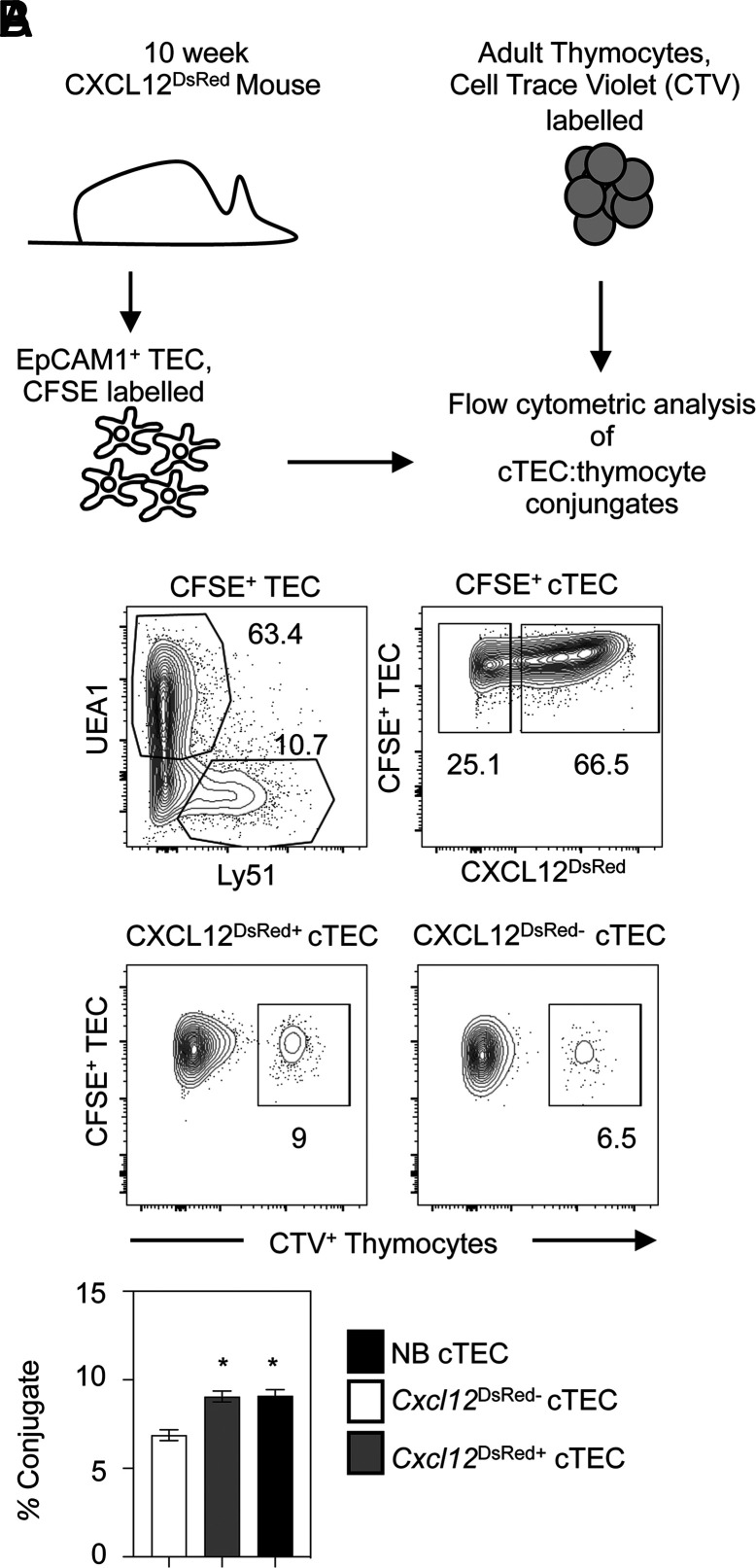
*Cxcl12^DsRed^*^−^ cTECs demonstrate an impaired capacity for thymocyte interactions. (**A**) The experimental approach used to study cTEC–thymocyte conjugate interactions using flow cytometry. (**B**) The gating approach used to compare the ability of *Cxcl12^DsRed+^* and *Cxcl12*^*DsRed*−^ cTECs to form conjugates with thymocytes. Successful thymocyte–cTEC conjugates appear as CFSE^+^TEC:CTV^+^ thymocyte events within *Cxcl12DsRed*^+^ and *Cxcl12DsRed*^−^ cTEC subsets. Quantitation of cTEC–thymocyte conjugate formation is also shown in (B), with comparison of conjugate formation with *Cxcl12DsRed*^+^ cTECs (gray bar), *Cxcl12^DsRed^*^−^ (white bar) cTECs, and neonatal cTECs (black bar). All data are representative of four individual experiments and four samples. The *p* values indicate significance using a Mann–Whitney nonparametric test: *p < 0.05. Error bars represent mean ± SEM.

The functional ability of cTECs is regulated by their expression of several key genes now known to be Foxn1 targets (18). Interestingly, a recent study (42) has shown that the formation of successful cellular interactions with thymocytes requires CXCL12 and DLL4, both of which are Foxn1 targets that are absent from *Cxcl12*^DsRed−^ cTECs. Given these differences between *Cxcl12*^DsRed+^ and *Cxcl12*^DsRed−^ cTECs, we wondered whether this may have functional consequences for their abilities to influence T cell development. To investigate this, we performed a flow cytometry–based cell conjugate assay where TEC–thymocyte interactions occur in a TCR-MHC–independent manner (35) to compare the ability of *Cxcl12*^DsRed+^ and *Cxcl12*^DsRed−^ cTECs to form successful TEC–thymocyte conjugates. In this study, purified EpCAM1^+^ TECs were FACS sorted from adult *Cxcl12*^DsRed^ mice, labeled with the fluorescent dye CFSE, and mixed with CellTrace Violet–labeled thymocytes at a ratio of 5:1 thymocytes:TEC (Fig. 7A). After centrifugation and 20-min incubation, pellets were gently disrupted, and conjugate formation was assessed by flow cytometry after gating on *Cxcl12*^DsRed+^ and *Cxcl12*^DsRed−^ cTECs within the total cTEC population (Fig. 7B). Although both *Cxcl12*^DsRed+^ and *Cxcl12*^DsRed−^ cTECs were capable of conjugate formation, we saw a significant decrease in conjugates formed from *Cxcl12*^DsRed−^ cTECs (Fig. 7B), suggesting *Cxcl12*^DsRed−^ cTECs may be less effective than their *Cxcl12*^DsRed+^ counterparts in influencing T cell development. Interestingly, when we compared the efficiency of TEC–conjugate formation using adult *Cxcl12*^DsRed+^ cTECs and neonatal cTECs, the latter being uniformly *Cxcl12*^DsRed+^ (Fig. 1C), we found them to be equally effective in mediating thymocyte interactions (Fig. 7B). Thus, the ability of *Cxcl12*^DsRed+^ cTECs to influence cortex-dependent thymocyte development may be consistent throughout the life course, and any changes in this process may occur as a result of the progressive emergence of *Cxcl12*^DsRed−^ cTECs.

## Discussion

Interactions between thymocytes and cTEC/mTEC populations support the intrathymic development and selection of αβT cells. Through examination of the cTEC compartment, we identified a developmentally regulated program of heterogeneity that occurs over the life course and is defined by loss of expression of Foxn1 and its downstream targets. Although our finding that all TECs arise from Foxn1-expressing cells is consistent with previous reports (20), what causes some cTECs to downregulate Foxn1, and Foxn1-dependent genes, is not known. Importantly, although Foxn1^−^ TECs have been described previously (43–45), multiple features, including their intrathymic positioning, transcriptomic profile, and intrathymic generation, have remained poorly understood. In this article, by identifying the gene profile of these cells, including their loss of a functionally important cTEC gene signature, we provide evidence they are transcriptionally distinct from their Foxn1-expressing counterparts. Moreover, the intrathymic positioning within the cortex of the cTEC subsets defined in this study, together with their regulation by CD4^−^CD8^−^, but not CD4^+^CD8^+^, thymocytes, extends our understanding of the complexity of the cTEC compartment and the mechanisms that control this. Indeed, because the appearance of cTECs that lack Foxn1 and its key target genes is regulated by thymocyte cross-talk, in particular events specific to CD4^−^CD8^−^ thymocytes, it may be that early stages of T cell development generate signals that cause loss of Foxn1, which then results in cTEC heterogeneity. Interestingly, analysis from birth up to 20 wk of age showed that the frequency of *Cxcl12*^DsRed−^ cTECs had plateaued by around 10 wk, which may indicate that turnover of *Cxcl12*^DsRed−^ cells takes place, rather than a process that results in their progressive accumulation during the life course.

The presence within the adult thymic cortex of cTECs that no longer express key genes regulating specific stages of thymocyte development raises multiple interesting scenarios. For example, it may be relevant to understanding progressive changes in thymus function under homeostatic conditions. In this study, because both *Cxcl12* and *Dll4* are important regulators of the β-selection checkpoint (46), absence of these genes in Foxn1^−^ cTECs may impact the ability of the thymus to support transition to the CD4^+^CD8^+^ stage. Also significant is that although *Psmb11*, the cTEC-specific gene encoding the thymoproteosome component β5t, is unique to cTEC (12), our data suggest that not all adult cTECs express transcripts of *Psmb11.* Thus, it may be the case that in the adult thymus, both *Psmb11*^+^ and *Psmb11*^−^ cTECs contribute to CD8^+^ SP selection, but they generate distinct αβTCR repertoires as a result of differences in the MHC I–bound self-peptides they can produce (thymoproteosome/β5t-dependent peptides for *Cxcl12*^DsRed+^ cTECs versus nonthymoproteosome/β5t-independent peptides for *Cxcl12*^DsRed−^ cTECs). In this article, it is important to note that β5t-deficient mice are still able to positively select some SP8^+^ thymocytes (47), a finding that may be consistent with the scenario that cTECs lacking *Psmb11* can to contribute to SP8 generation in normal mice. Alternatively, adult *Foxn1*^−^ cTECs that lack *Psmb11* may be incapable of positive selection because of other functional defects, such as a failure to interact with CD4^+^CD8^+^ thymocytes. Although it is interesting to note that *Cxcl12*^DsRed−^ cTECs express significantly lower levels of MHC I relative to their *Cxcl12*^DsRed+^ counterparts, and form fewer cell–cell conjugates with thymocytes, further studies are required to examine the functional properties of the cTEC subsets described in this article. Relevant to this, our attempts to compare the functional abilities of FACS-sorted *Cxcl12*^DsRed+^ and *Cxcl12*^DsRed−^ cTECs from adult mice in reaggregate thymus organ cultures were unsuccessful. In this study, intact three-dimensional structures consistently failed to form when using TECs isolated from adult mice, which is in contrast with the efficient generation of intact reaggregate thymus organ culture from embryonic TECs (48, 49). The reasons for the inability of adult TECs to effectively form reaggregate thymus organ culture under conditions that support embryonic TEC reaggregation are not clear. However, it is interesting to note that early studies on the capacity of embryonic tissues to undergo effective reaggregation attributed this to their ability to undergo what was termed “inductive interactions” (50), which may be missing from adult TECs. Whatever the case, further studies are required to compare the functional capacity of cTEC subsets described in this article, which would also benefit from the creation of improved experimental systems to study adult TEC functions in vitro.

Beyond directly influencing specific stages of thymocyte development, *Cxcl12*^DsRed−^ cTECs may also play a role in physically supporting the epithelial scaffold within the thymus cortex, a possibility raised recently in the context of the presence of FOXN1^−^ TECs in thymus (44). Such a possibility may be compatible with our finding that *Cxcl12*^DsRed−^ cTECs are interspersed in the cortex alongside *Cxcl12*^DsRed+^ cells. A final possibility is that alongside loss of Foxn1-mediated functional properties, *Cxcl12*^DsRed−^ cTECs acquire new functional features that are important in adult thymus cortex organization and/or function. Again, further examination requires approaches to directly assess the functional properties of defined cTEC subsets.

In summary, we show that the Ly51^+^UEA1^−^ cTEC compartment undergoes developmentally regulated changes in its cellular makeup that are driven by interactions with the maturation of immature CD4^−^CD8^−^ thymocytes. We identify the emergence of a cTEC subset that retains its Ly51^+^UEA1^−^ phenotype and positioning within the cortex but has ceased to express FOXN1, resulting in the lack of expression of key FOXN1 target genes that define the functional properties of cTECs. These findings demonstrate the emerging complexity of the thymic cortex and will aid in future studies that examine the role of this intrathymic site in thymocyte development.

## Supplementary Material

Data Supplement
